# Neoadjuvant targeted therapy versus targeted combined with chemotherapy for resectable EGFR-mutant non–small cell lung cancer: a retrospective controlled real-world study

**DOI:** 10.3389/fonc.2024.1349300

**Published:** 2024-07-16

**Authors:** Weipeng Shao, Zhan Liu, Bobo Li, Feng Chen, Jie Liu, Hui Li, Hongbo Guo

**Affiliations:** Department of Thoracic Surgical Ward II, Shandong Cancer Hospital and Institute, Shandong First Medical University and Shandong Academy of Medical Sciences, Jinan, Shandong, China

**Keywords:** NSCLC, EGFR-mutation, neoadjuvant targeted therapy, neoadjuvant targeted plus chemotherapy, MPR

## Abstract

**Background:**

This study aimed to assess the role and effect of neoadjuvant targeted therapy (TT) versus targeted combined with chemotherapy (TC) for resectable EGFR-mutant non–small cell lung cancer (NSCLC).

**Methods:**

Between March 2021 and June 2023, 20 patients with stage IA3-IIIB NSCLC were enrolled in the study. Eleven patients received EGFR-TKIs in the TT group, while nine patients received EGFR-TKIs and two cycles of cisplatin-based doublet chemotherapy (TC group). We compare the differences between the two groups through the following variables, including age, sex, surgical approach, postoperative complications, neoadjuvant therapy adverse events, complete response (CR), partial response (PR), stable disease (SD), progressive disease (PD), objective response rate (ORR), major pathologic response (MPR), and pathologic complete response (pCR).

**Results:**

Patients were predominantly female (75%) and never-smokers (95%). The average age was 59.2 years (range 46-79 years). Fifty-five percent harbored an exon 19 EGFR mutation and 45% an exon 21 mutation. The average targeted drug dosing time was 2.91 ± 1.7 (range 1-6) months in the TT group and 3.56 ± 3.54 (range 1-12) months in the TC group (P=0.598). The most common side effects were rash and diarrhea. No grade 5 events with neoadjuvant therapy were observed. The rate of R0 resection was 100% in all patients. Among the 11 patients in the TT group, 6 achieved a PR and 5 had SD, resulting in an ORR of 54.5%. Among the 9 patients in the TC group, 6 had PR and the remaining 3 had SD, resulting in an ORR of 66.6%. one patient (11.1%) in the TC group achieved pCR, while no patients in the TT group achieved pCR (P = 0.142). Two patients (18.2%) in the TT group reached MPR, and 2 patients (22.2%) in the TC group reached MPR (P = 0.257). The overall clinical downstage rate is 60%. Only 9 (45%) cases of yield clinical TNM (ycTNM) were consistent with yield pathologic TNM (ypTNM).

**Conclusion:**

Results from this retrospective controlled research indicate that the neoadjuvant TT group is likely to be more effective outcomes and has safer profile in patients with EGFR-positive NSCLC than the neoadjuvant TC group. However, our results need to be validated in a multicenter, large sample prospective study.

## Introduction

Lung cancer is the leading cause of cancer death in China and the world (1, 2). Previously, treatment options for potentially resectable patients with non-small cell lung cancer (NSCLC) include neoadjuvant therapy with cisplatin or carboplatin; and subsequent adjuvant chemotherapy and/or radiotherapy to prevent rapid recurrence (3). However, this benefit has been questioned (4). With the advent of Checkmate-816, immunotherapy alone or in combination with chemotherapy has created a wave of new research in the neoadjuvant setting (5). Clinical evidence has shown that patients with advanced, EGFRmutant NSCLC derive little or no benefit from cancer immunotherapy combining with or without targeted therapies (6, 7). In the past few decades, the increasing knowledge of cancer biology has led to the introduction of new targeted therapies for lung cancer (2). These new therapies target specific cancer processes and hence have the potential to be more effective and less toxic (8). Adjuvant targeted therapies (epidermal growth factor receptor (EGFR) tyrosine kinase inhibitor (TKI)) have revolutionized the NSCLC care in the advanced disease setting (9). The recommended first-line treatment for patients with oncogene-addicted advanced NSCLC is targeted therapies. Targeted therapies could be beneficial in the neoadjuvant setting for this group of patients (10, 11). There are no completed targeted therapy clinical trials and no current neoadjuvant standard of care in the management of EGFR mutation-positive (EGFRm) NSCLC, but several studies (NCT01833572, NCT01217619, EMERGING-CTONG 1103, NEOS, and NCT03433469) are now recruiting (12–14). Considering the advantages of preoperative targeted therapy and the disadvantages of preoperative neoadjuvant chemotherapy, can we consider conducting separate neoadjuvant targeted therapy for this type of patient? Therefore, we conducted a retrospective controlled study of EGFR-TKIs combined with chemotherapy versus EGFR-TKIs alone as neoadjuvant therapy in the treatment of EGFR-mutation positive resectable NSCLC.

## Patients and methods

Twenty treatment-naive patients with stageIA3-IIIB NSCLC were enrolled in the study from March 2021 to June 2023 at the Shandong Cancer Hospital and Institute. Inclusion criteria included: 1) age 18 years or older; 2) no previous treatment for lung cancer; 3) Eastern Cooperative Oncology Group (ECOG) performance status (PS) of 0 or 1 (15); 4) pathologically confirmed as adenocarcinoma; 5) with EGFR mutations in exon 19 or 21; 6) underwent surgery; and 7) neoadjuvant targeted therapy or targeted combined with chemotherapy. Exclusion criteria included: 1) history of malignancies in the past 5 years; 2) previous local radiotherapy or any systemic antitumor therapy;3) unstable systemic disease; and 4) history or current diagnosis of interstitial lung disease (ILD).

The study was conducted in accordance with the Declaration of Helsinki and approved by the ethics committee of the Shandong Cancer Hospital and Research Institute (No. SDTHEC2024002005).

Eleven patients received EGFR-TKIs as neoadjuvant therapy (TT group), while nine patients received EGFR-TKIs and two cycles of platinum-based doublet chemotherapy in combination with docetaxel or pemetrexed (TC group). Platinum-based drugs included carboplatin, with an area under the curve of 5, or cisplatin 25 mg/m2 on days 1–3. Pemetrexed (500 mg/m2) was then administered. Patients received 1–3 doses of preoperative chemotherapy every three weeks, and the average usage cycle was two in the TC groups. The target drugs included osimertinib, gefitinib, almonertinib, afatinib, furmonertinib, icotinib.

Patients underwent chest Computed Tomography (CT), abdomen CT, and Emission CT (ECT) scans; brain magnetic resonance imaging (MRI), or Positron Emission Tomography CT (PET-CT); bronchoscope or Endobronchial ultrasound-guided transbronchial needle aspiration (EBUS-TBNA); and examinations of cardiac function. In our routine clinical practice, simplified radiological evaluation (enhanced CT or PET-CT) was more common for N-stage patients. Only a few patients have undergone bronchoscopy or EBUS-TBNA.

The stages of the primary pulmonary tumor (T), lymph node (N), and metastasis (M) were evaluated based on the American Joint Committee on Cancer 8th edition TNM staging system for NSCLC (16). After neoadjuvant therapy, enhanced CT was performed to observe the response of the tumor to drugs, and the tumor size was evaluated according to RECIST 1.1 (response evaluation criteria in solid tumors version 1.1) (17). The evaluation of the target lesions was divided into complete response (CR), partial response (PR), stable disease (SD), progressive disease (PD), and objective response (OR) including PR+CR. All the patients were monitored for adverse events, according to the National Cancer Institute Common Terminology Criteria for Adverse Events version 5.0 (NCI-CTCAE 5.0) (18). Major pathologic response (MPR) was defined as residual viable tumor cells less than 10%, and pathologic complete response (pCR) was defined as no residual viable tumor (19).

### Statistical analysis

Categoric variables were expressed as numbers or percentages and evaluated with λ^2^ or Fisher’s exact test. Continuous data were presented as mean ± SD and were compared using the two-sample Student t-test. All P-values were reported by 2-sided analyses, and the statistical significance level was set at less than 0.05. Statistical analyses were performed with SPSS 24.0 software (SPSS, Inc., Chicago, IL, USA) and R version 4.1 (R Foundation for Statistical Computing).

## Results

### Patient characteristics

**Figure 1:**
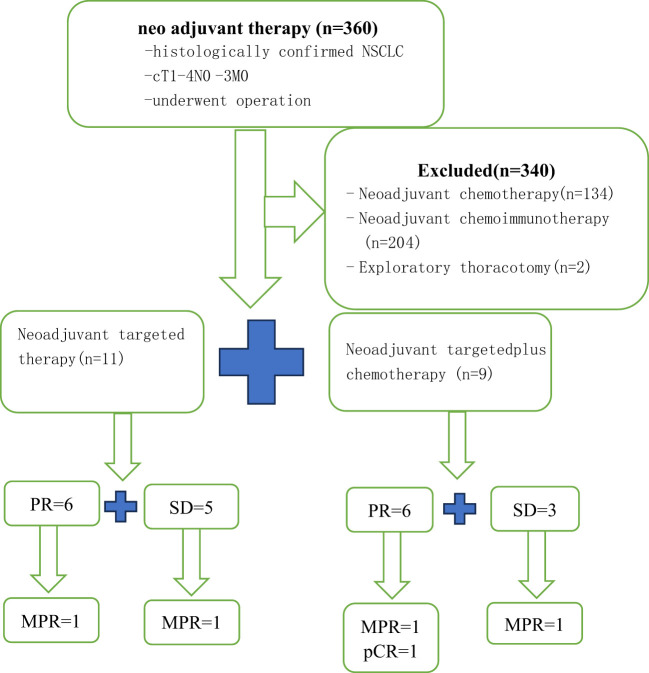
Flow diagram of study design.

Twenty NSCLC patients were recruited (**Figure 1**). Patients were predominantly female (75%) and never-smokers (95%), only one patient had ever-smoking. The average age was 59.2 years (range 46-79 years). Fifty-five percent had an exon 19 EGFR mutation and 45% had an exon 21 mutation. Most patients were stage clinical T2(cT2) NSCLC (TT group, 36.3%; TC group, 44.4%). The proportion of patients with cN2(55%) and cIIIA (55%) stages was the highest. Age, gender, smoking status, EGFR status, ECOG PS, dosing time, and clinical stage were balanced between arms. The average dosing time was 2.91 ± 1.7 (range 1-6) months in the TT group and 3.56 ± 3.54 (range 1-12) months in the TC group (P=0.598). The patient demographics and clinical characteristics are summarized in **Table 1**.

**Table 1:** Patient demographics and clinical characteristics.

characteristic		total	TKI	TKI+Chemotehrapy	p-value
			11	9	
EGFR status	Exon 19 deletion	11	6	5	0.964
	L858R 21	9	5	4	
ECOG PS	0	13	8	5	0.423
	1	7	3	4	
Sex	male	5	2	3	0.436
	female	15	9	6	
smoking status	ever	1	1	0	0.353
	never	19	10	9	
cT	1	5	3	2	0.528
	2	8	4	4	
	3	2	2	0	
	4	5	2	3	
cN	0	4	2	2	0.067
	1	4	0	4	
	2	11	8	3	
	3	1	1	0	
cTNM	IA3	1	1	0	0.11
	IB	2	0	2	
	IIA	1	1	0	
	IIB	1	0	1	
	IIIA	11	5	6	
	IIIB	4	4	0	
age, years			60.91 ± 8.50	57.11 ± 8.08	0.323
dosing time, months			2.91 ± 1.7	3.56 ± 3.54	0.598

EGFR, epidermal growth factor receptor; TKI, tyrosine kinase inhibitor; ECOG PS, Eastern Cooperative Oncology Group performance status.

### Safety and tolerability

Overall, the two neoadjuvant treatment regimens were well tolerated. The most common side effects were rash and diarrhea. No grade 5 events with neoadjuvant therapy were observed. As shown in the **Table 2**, most patients underwent video-assisted thoracic surgery (VATS) (85%) and lobectomy (95%). No patient converted to pneumonectomy because of the complexity of the operation. The rate of R0 resection was 100% in all patients. The average operation time was 121.36 ± 62.45 minutes in the TT group and 91.11 ± 20.28 minutes in the TC group. Although the time of operation varies greatly, it is not statistically significant (p=0.155). We traced the operation time data and found that in the TT group, there were 2 cases with longer operation times (240min and 180min, respectively), which resulted in longer mean operation times. There were 2 cases in the TT group (postoperative chylothorax and hydrothorax). No perioperative mortality was found in either arm. There was no significant difference between the two arms. The follow-up time in the TC group (25 ± 5.15 months) was significantly longer than that in the TT group (18 ± 5.16 months) (P=0.007). One case in the TC group occurred brain metastasis 19 months after being diagnosed with lung cancer. After follow-up, further treatment is still underway.

**Table 2:** Patient surgical and postoperative characteristics.

characteristic		total	TKI	TKI+Chemotehrapy	p-value
operation methods	lobectomy	19	11	8	0.257
	bilobectomy	1	0	1	
surgery	thoractomy	3	1	2	0.413
	VATS	17	10	7	
complication	no	18	9	9	0.178
	yes	2	2	0	
operation time (min)			121.36 ± 62.45	91.11 ± 20.28	0.155
follow-up time (months)			18 ± 5.16	25 ± 5.15	0.007
discharge time (days)			5.91 ± 1.70	6.78 ± 2.59	0.378
status	alive	19	10	9	0.353
	death	1	1	0	

TKI, tyrosine kinase inhibitor.

### Efficacy

#### Clinical responses to neoadjuvant therapies

**Figure 2:**
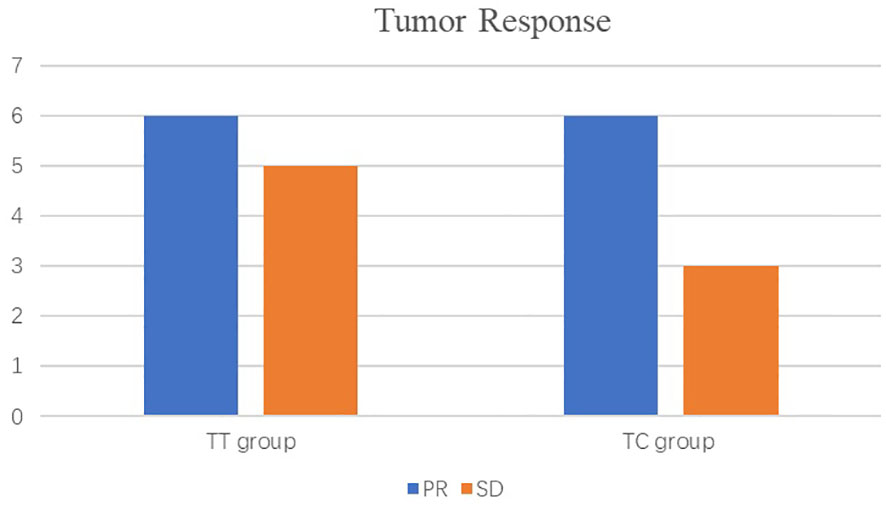
Clinical responses to neoadjuvant treatment.

**Figure 3:**
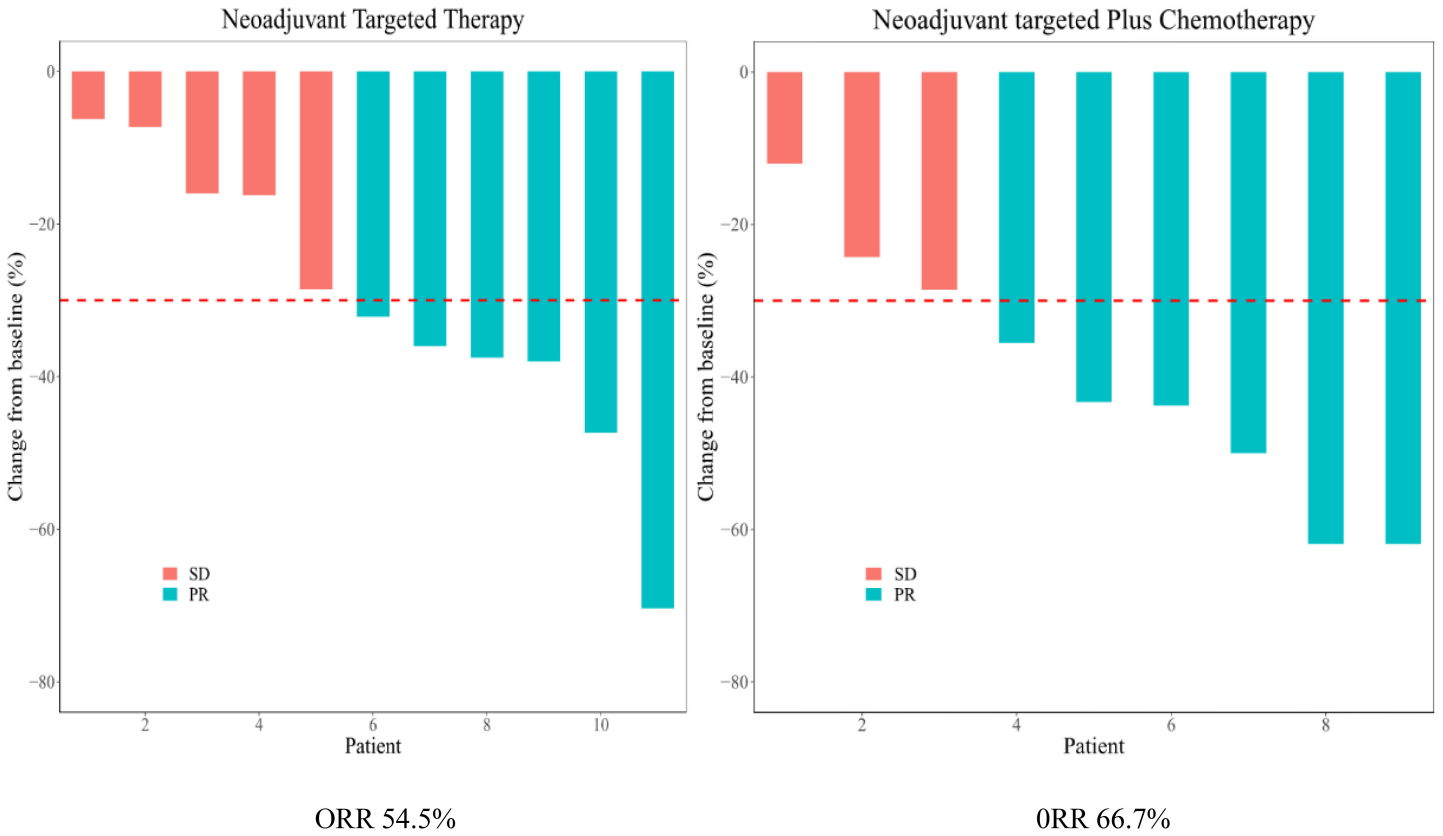
Waterfall plot of each arm.

As shown in the histogram (**Figure 2**) waterfall plot (**Figure 3**), among the 11 patients in the TT group, 6 acquired a PR and 5 had SD, resulting in an ORR of 54.5%. Of the 9 patients in the TC group, 6 had PR, and the remaining 3 had SD, resulting in an ORR of 66.6%. Our data show that patients in the TC group had a better ORR than those in the TT group, although the difference was not statistically significant (P=0.582). After neoadjuvant treatment, the overall clinical downstage rate is 50%. The TT group and TC group were 54.5% and 44.4% (P=0.653), respectively (**Table 3**).

**Table 3:** Evaluation of neoadjuvant therapy efficacy.

characteristic		total	TKI	TKI+Chemotehrapy	p-value
			11	9	
ycT	1	12	6	6	0.019
	2	5	5	0	
	3	3	0	3	
ycN	0	7	3	4	0.249
	1	7	3	4	
	2	6	5	1	
ycTNM	IA1	1	0	1	0.426
	IA2	2	1	1	
	IA3	1	0	2	
	1B	1	1	0	
	IIA	1	1	0	
	IIB	4	3	1	
	IIIA	9	5	4	
ORR	yes	12	6	6	0.582
	no	8	5	3	
PR	yes	12	6	6	0.582
	no	8	5	3	
SD	yes	8	5	3	0.582
	no	12	6	6	
cDownstage	yes	10	6	4	0.653
	no	10	5	5	
ypT	0	1	0	1	0.082
	IA	10	3	7	
	IB	5	4	1	
	IC	1	1	0	
	2A	3	3	0	
ypN	0	10	3	7	0.073
	1	1	1	0	
	2	9	7	2	
ypTNM	0	1	0	1	0.105
	IA1	8	2	6	
	IA2	1	1	0	
	IIB	1	1	0	
	IIIA	9	7	2	
pDownstage	yes	12	5	7	0.142
	no	8	6	2	
ycTNM=ypTNM	yes	9	7	2	0.064
	no	11	4	7	
MPR	yes	4	2	2	0.822
	no	16	9	7	
CPR	yes	1	0	1	0.257
	no	19	11	8	

TKI, tyrosine kinase inhibitor; ORR, objective response rate; PR, partial response; SD, stable disease; MPR, major pathologic response; pCR, pathologic complete response; ycTNM, yield clinical TNM; ypTNM, yield pathological TNM.

#### Pathological responses to neoadjuvant therapies

In the postoperative pathological analysis (**Table 3**), one patient (11.1%) in the TC group achieved pCR, while no patients in the TT group achieved pCR. The difference was not statistically significant (P = 0.142). Two patients (18.2%) in the TT group reached MPR, and 2 patients (22.2%) in the TC group reached MPR, the difference was not statistically significant (P = 0.257). From this, we can conclude that targeted drugs alone may achieve the effect of targeted plus chemotherapy. The overall clinical downstage rate is 60%. The TT group and TC group were 45.5% and 77.8% (P=0.142), respectively. We compared the yield clinical TNM (ycTNM) and yield pathological TNM (ypTNM) stages of each patient separately. Only 9 (45%) cases of ycTNM were consistent with ypTNM. The TT group and TC group have 7 (63.6%) and 2 (22.2%) (P=0.064), respectively. The remaining 11 patients had inconsistent staging, eight patients achieved better downstage than ycTNM. The TT group and TC group have 2 and 6, respectively. Both the TT group and TC group have two up-stage. The difference between post-treatment and postoperative stages reminds us of the limitations of the imaging assessment of staging.

## Discussion

In a meta-analysis of patients with resectable NSCLC, neoadjuvant chemotherapy was found to improve 5-year overall survival (OS) by 5% compared with surgery alone (3). Therefore, there is a need for new and effective treatments to reduce the recurrence of disease, prolong the survival time, and improve the cure rates. Given the success of target therapy in the advanced disease setting, there is increasing research on neoadjuvant targeted therapy for mutation-driven resectable NSCLC (11, 13). Is targeted therapy combined with chemotherapy more effective as neoadjuvant therapy? Therefore, we conducted this retrospective controlled study.

In this study, patients who received TT or TC had similar clinical and pathological effects. Lara-Guerra H et al. demonstrated that gefitinib was a generally safe and feasible regimen for neoadjuvant therapy in unselected patients with stage I NSCLC, with a PR of 11%(NCT00188617) (13). Zhang Y et al. found that neoadjuvant gefitinib was a viable treatment option for stage II-IIIA NSCLC patients with EGFR-mutant, ORR, and MPR were 54.5% and 24.2%, respectively (NCT01833572) (11). Xiong L et al. reported that erlotinib resulted in a higher ORR (67%vs.19%) and pathologic response rate (67%vs.38%) than platinum-based adjuvant chemotherapy (PBAC) (NCT01217619) (20). The EMERGING-CTONG 1103 study found that the ORR for neoadjuvant erlotinib versus chemotherapy was 54.1% versus 34.3%. The MPR was 9.7% and 0%, respectively. No pCR was identified in either arm (10). The above studies were mostly single-arm studies of neoadjuvant TT or control studies of neoadjuvant TT compared to neoadjuvant chemotherapy, with varying clinical and pathological results obtained. Few studies directly compare neoadjuvant TT and TC. Combined with our results, or neoadjuvant targeted therapy alone, we could achieve the expected results. The reason why we propose this viewpoint is inspired by postoperative adjuvant therapy. For example, Tetsuya Isaka et al. showed that that patients with EGFR mutations (–) who received PBAC had better OS than those who did not receive PBAC, although EGFR mutation (+) patients who did and did not receive PBAC had no difference in OS. They concluded that neoadjuvant chemotherapy might not be necessary for EGFR mutation (+) patients with pathological stage II/III NSCLC (21). In another study from Japan, Yasuhiro Tsutani et al. evaluated the role and effect of adjuvant chemotherapy based on EGFR mutation status in patients with stage I lung adenocarcinoma. For patients with EGFR mutation (+), who received adjuvant chemotherapy or not, there was no significant difference in the 5-year recurrence-free survival (RFS) (74.3% vs 80.5%, P=0.573) and OS (91.7% vs 97.8%, P=0.183). For patients with EGFR mutation (-/unknown), received adjuvant chemotherapy or not, there were significant differences in the 5-year RFS (88.4% vs 63.6%, P=0.001) and OS (93.2% vs 77.9%, P=0.008) (22). There is another study from China, Wenyu Zhai et al. demonstrated that adjuvant chemotherapy was associated with improved OS and DFS outcomes in patients with EGFR mutation (–), but not benefit with EGFR mutation (+) (23). Harry B. Lengel et al. proved that preoperative targeted therapy was well tolerated and associated with good outcomes, regardless of induction chemotherapy (24). The ongoing clinical trials (NCT04470076, NCT04351555, NCT05011487, NCT05132985, NCT05430802) might have certain reference opinions for determining the choice of neoadjuvant treatment options (12).

A Phase II study of preoperative gefitinib in clinical stage I NSCLC showed that 83% of patients had consistent clinical and pathological staging (13). Ye Ning et al. retrospectively evaluated the survival rate of 10 patients with advanced NSCLC who underwent salvage surgery after EGFR-TKI neoadjuvant therapy and found that 70% of patients have consistent clinical and pathological staging. In the remaining 3 cases, 1 case was downstaged, and the other 2 cases were upstaged (25). NEOS is a prospective, multicenter, single-arm study designed to evaluate the efficacy and safety of osimertinib as a neoadjuvant treatment in resectable EGFR mutation (+) lung adenocarcinoma. The disease control rate (DCR) was 100% (15/15), 53.3% (8/15) of patients had a pathological decline, and 42.9% (3/7) of the patients with confirmed N2 lymph nodes experienced downstaging to N0 disease after receiving neoadjuvant osimertinib (26).

The above studies are all single-arm studies. In the present study, there was a certain degree of decline in both the TT group and the TC group. However, the concordance rate of the clinical stage and postoperative stage in the TT group was higher than that in the TC group. In the TC group, after the neoadjuvant target was combined with chemotherapy, to some extent, the tumor shrank more significantly than that measured on CT. Compared to the TT group, chemotherapy might affect tumor shrinkage, but further molecular-level research is needed to determine the specific mechanism. Even if the tumor was somewhat responsive to drug, it still takes time for the necrotic lesion to absorb after targeted therapy, resulting in a reduction in the diameter measured by CT. This results in inconsistency between clinical remission and pathological outcome.

Compared with TC, TT has unique safety and tolerability in the neoadjuvant setting. It is important to consider whether there is any toxicity that may delay or prevent the efficacy of surgery during neoadjuvant therapy. Compared to neoadjuvant chemotherapy, the use of EGFR inhibitors has fewer severe respiratory adverse events (including pneumonitis and interstitial lung disease), which could limit the use of neoadjuvant therapy (27).

In our study, no grade 5 events were observed. The results demonstrate that most patients can tolerate these two schemes. Most patients underwent VATS and lobectomy. No patient received a pneumonectomy. The rate of R0 resection was 100%. Judging from these data, the two schemes have little impact on the operation. A narrative review similar to our results suggests that neoadjuvant target therapy is well tolerated in resectable NSCLC patients, with all patients undergoing surgery without delay or major complications (27, 28). Delays in surgery due to adverse events from oncology treatment, limitations in diagnostic services provided, and the possibility of progression during treatment may have challenged to the selection of neoadjuvant chemotherapy. It may be that our sample size is too small to show this difference between the two groups. The ongoing clinical trials will provide further information on the safety and tolerability of a wider range of TT for neoadjuvant treatment of resectable NSCLC.

The research on the neoadjuvant TT or TC is still early and less, and the optimal course of treatment is not yet known. Because of our retrospective study, it is difficult to control the preoperative medication time, which ranges from 1 month to 12 months. We also have no clear recommendations for the medication cycle. Perhaps according to the evaluation of CT (RECIST 1.1) after-treatment is a better choice.

There were several limitations to this research. Firstly, patient selection bias may have existed due to the retrospective nature of this real-world study. Secondly, given the limited number of patients in our cohort, larger multi-center or even prospective studies are necessary to confirm our findings. Finally, our results might be influenced by tumor characteristics and surgeon’s techniques, experience, and preferences.

## Conclusion

To our knowledge, few studies have conducted real-world studies on neoadjuvant targeted therapy versus targeted combined with chemotherapy for resectable EGFR-Mutant NSCLC. Results from this retrospective controlled research indicated that the neoadjuvant TT group was likely to be more effective outcomes and has safer profile in patients with EGFR-positive NSCLC than the TC group. Therefore, we recommend further investigation through a prospective study to validate the findings of this retrospective analysis.

## Data availability statement

The original contributions presented in the study are included in the article/supplementary files, further inquiries can be directed to Weipeng Shao, shaoweipeng2015@163.com.

## Ethics statement

This study was approved by the institutional review board of Shandong Cancer Hospital and Institute (SDTHEC2024002005). The requirement for written informed consent from individual patients for this retrospective analysis was waived by the institutional board.

## Author contributions

WS: Conceptualization, Data curation, Formal analysis, Investigation, Methodology, Project administration, Resources, Software, Validation, Visualization, Writing – original draft, Writing – review & editing. BL: Formal analysis, Investigation, Methodology, Project administration, Resources, Supervision, Writing – original draft. ZL: Conceptualization, Data curation, Formal analysis, Software, Writing – review & editing. FC: Investigation, Methodology, Project administration, Resources, Writing – review & editing. JL: Formal analysis, Methodology, Supervision, Writing – review & editing. HL: Formal analysis, Software, Supervision, Writing – review & editing. HG: Conceptualization, Investigation, Resources, Supervision, Validation, Writing – review & editing.

## References

[1] Chen W Zheng R Baade PD Zhang S Zeng H Bray F. Cancer statistics in China, 2015. CA Cancer J Clin. (2016) 66:115–32. doi: 10.3322/caac.21338 26808342

[2] Siegel RL Giaquinto AN Jemal A. Cancer statistics, 2024. CA Cancer J Clin. (2024) 74:12–49. doi: 10.3322/caac.21820 38230766

[3] Group NM-aC. Preoperative chemotherapy for non-small-cell lung cancer: a systematic review and meta-analysis of individual participant data. Lancet. (2014) 383:1561–71. doi: 10.1016/S0140-6736(13)62159-5 PMC4022989 24576776

[4] Pisters KMW Vallieres E Crowley JJ Franklin WA Bunn PA Ginsberg RJ. Surgery with or without preoperative paclitaxel and carboplatin in early-stage non–small-cell lung cancer: southwest oncology group trial S9900, an intergroup, randomized, phase III trial. J Clin Oncol. (2010) 28:1843–9. doi: 10.1200/JCO.2009.26.1685 PMC2860367 20231678

[5] Patrick MF Jonathan S Shun L Mariano P Tetsuya M Mark MA. Neoadjuvant nivolumab plus chemotherapy in resectable lung cancer. N Engl J Med. (2022) 386:1973–85. doi: 10.1056/NEJMoa2202170 PMC9844511 35403841

[6] Lisberg A Cummings A Goldman JW Bornazyan K Reese N Wang T. A phase II study of pembrolizumab in EGFR-mutant, PD-L1+, tyrosine kinase inhibitor (TKI) nave patients with advanced NSCLC. J Thorac Oncol Off Publ Int Assoc Study Lung Cancer. (2018) 12:S1805. 10.1016/j.jtho.2018.03.035 PMC6063769 29874546

[7] Gainor JF Shaw AT Sequist LV Fu X Azzoli CG Piotrowska Z. EGFR mutations and ALK rearrangements are associated with low response rates to PD-1 pathway blockade in non–small cell lung cancer: a retrospective analysis. Clin Cancer Res Off J Am Assoc Cancer Res. (2016) 22(18):4585–93. doi: 10.1158/1078-0432.CCR-15-3101 PMC5026567 27225694

[8] Hanahan D Weinberg Robert A. Hallmarks of cancer: the next generation. Cell. (2011) 144:646–74. doi: 10.1016/j.cell.2011.02.013 21376230

[9] White MN Piotrowska Z Stirling K Liu SV Banwait MK Cunanan K. Combining osimertinib with chemotherapy in EGFR-mutant NSCLC at progression. Clin Lung Cancer. (2021) 22:201–9. doi: 10.1016/j.cllc.2021.01.010 PMC8205932 33610453

[10] Zhong WZ Chen KN Chen C Gu CD Wu YL. Erlotinib versus gemcitabine plus cisplatin as neoadjuvant treatment of stage IIIA-N2 EGFR -mutant non–small-cell lung cancer (EMERGING-CTONG 1103): A randomized phase II study. J Clin Oncol. (2019) 37:2235–45. doi: 10.1200/JCO.19.00075 31194613

[11] Zhang Y Fu F Hu H Wang S Chen H. Gefitinib as neoadjuvant therapy for resectable stage II-IIIA non–small cell lung cancer: A phase II study. J Thorac Cardiovasc Surgery. (2021) 161:434–42.e2. doi: 10.1016/j.jtcvs.2020.02.131 32340810

[12] Tsuboi M Weder W Escriu C Blakely C Chaft JE. Neoadjuvant osimertinib with/without chemotherapy versus chemotherapy alone for EGFR -mutated resectable non-small-cell lung cancer: NeoADAURA. Future Oncol. (2021) 17:4045–55. doi: 10.2217/fon-2021-0549 PMC8530153 34278827

[13] Lara-Guerra H Waddell TK Salvarrey MA Joshua AM Chung CT Paul N. Phase II study of preoperative gefitinib in clinical stage I non-small-cell lung cancer. J Clin Oncol Off J Am Soc Clin Oncol. (2009) 27:6229–36. doi: 10.1200/JCO.2009.22.3370 19884551

[14] Lee JM McNamee CJ Toloza E Negrao MV Lin J Shum E. Neoadjuvant targeted therapy in resectable NSCLC: current and future perspectives. J Thorac Oncol. (2023) 18:1458–77. doi: 10.1016/j.jtho.2023.07.006 PMC11040203 37451404

[15] Oken M Creech R Tormey D Horton J Davis T Mcfadden E. Toxicity and response criteria of the Eastern Cooperative Oncology Group. Am J Clin Oncol: Cancer Clin Trials. (1982) 5(6):649–656. doi: 10.1097/00000421-198212000-00014 7165009

[16] Detterbeck FC Boffa DJ Kim AW Tanoue LT. The eighth edition lung cancer stage classification. Chest. (2017) 151:193–203. doi: 10.1016/j.chest.2016.10.010 27780786

[17] Yang Z Wang S Yang H Jiang Y Zhu L Zheng B. Treatment patterns and clinical outcomes of patients with resectable non-small cell lung cancer receiving neoadjuvant immunochemotherapy: a large-scale, multicenter, real-world study (NeoR-World). J Thorac Cardiovasc Surg. (2024). S0022-5223(24)00111-9. doi: 10.1016/j.jtcvs.2024.02.006 38342430

[18] Freites-Martinez A Santana N Arias-Santiago S Viera A. Using the Common Terminology Criteria for Adverse Events (CTCAE – Version 5.0) to Evaluate the Severity of Adverse Events of Anticancer Therapies. Actas Dermo-Sifiliográficas. (2020) 112(1). doi: 10.1016/j.adengl.2019.05.021 32891586

[19] Travis W Dacic S Wistuba I Sholl L Adusumilli P Bubendorf L. IASLC multidisciplinary recommendations for pathologic assessment of lung cancer resection specimens after neoadjuvant therapy. J Thorac Oncol. (2020) 15:709–40. doi: 10.1016/j.jtho.2020.01.005 PMC8173999 32004713

[20] Xiong L Lou Y Bai H Li R Xia J Fang W. Efficacy of erlotinib as neoadjuvant regimen in EGFR-mutant locally advanced non-small cell lung cancer patients. J Int Med Res. (2020) 48:300060519887275. doi: 10.1177/0300060519887275 31885349 PMC7607055

[21] Isaka T Ito H Nakayama H Yokose T Katayama K Yamada K. Efficacy of platinum-based adjuvant chemotherapy on prognosis of pathological stage II/III lung adenocarcinoma based on EGFR mutation status: a propensity score matching analysis. Mol Diagn Ther. (2019) 23:657–65. doi: 10.1007/s40291-019-00419-9 31347029

[22] Tsutani Y Ito M Shimada Y Ito H Ikeda N Nakayama H. The impact of epidermal growth factor receptor mutation status on adjuvant chemotherapy for patients with high-risk stage I lung adenocarcinoma. J Thorac Cardiovasc Surg. (2022) 164:1306–15.e4. doi: 10.1016/j.jtcvs.2022.01.025 35181001

[23] Zhai W Duan F Li D Yan Q Dai S Zhang B. Risk stratification and adjuvant chemotherapy after radical resection based on the clinical risk scores of patients with stage IB-IIA non-small cell lung cancer. Eur J Surg Oncol. (2022) 48:752–60. doi: 10.1016/j.ejso.2021.09.023 34620508

[24] Lengel HB Zheng J Tan KS Liu CC Park BJ Rocco G. Clinicopathologic outcomes of preoperative targeted therapy in patients with clinical stage I to III non-small cell lung cancer. J Thorac Cardiovasc Surg. (2023) 165:1682–93.e3. doi: 10.1016/j.jtcvs.2022.10.056 36528430 PMC10085825

[25] Ning Y Bao M Yan X Xie D Jiang G. Surgery for advanced non-small cell lung cancer patient after epidermal growth factor receptor tyrosine kinase inhibitor neoadjuvant therapy. Ann Trans Med. (2018) 6:407. doi: 10.21037/atm PMC6230859 30498734

[26] Lyu C Fang W Ma H Wang J Yang Y. Osimertinib as neoadjuvant treatment for resectable stage II-IIIB EGFR mutant lung adenocarcinoma (NEOS). J Clin Oncol. (2021) 39:8524–. doi: 10.1200/JCO.2021.39.15_suppl.8524

[27] Marjanski T Dziedzic R Kowalczyk A Rzyman W. Safety of surgery after neoadjuvant targeted therapies in non-small cell lung cancer: a narrative review. Int J Mol Sci. (2021) 22:12244. doi: 10.3390/ijms222212244 34830123 PMC8622767

[28] Stencel K Chmielewska I Milanowski J Ramlau R. Non-small-cell lung cancer: new rare targets-new targeted therapies-state of the art and future directions. Cancers (Basel). (2021) 13:1829. doi: 10.3390/cancers13081829 33921237 PMC8070470

